# Nationwide trends in the use of ADHD medications in the period 2006–2022: a study from the Norwegian prescription database

**DOI:** 10.1186/s12888-024-06199-9

**Published:** 2024-11-05

**Authors:** Ingeborg Hartz, Nils Henry Haugen Madsstuen, Per Normann Andersen, Marte Handal, Ingvild Odsbu

**Affiliations:** 1 Department of Research and Innovation, Innlandet Hospital Trust, Postbox 104, Brumunddal, 2381 Norway; 2 Department of Chronic Diseases, Norwegian Institute of Public Health, Postbox 222 Skøyen, Oslo, 0213 Norway; 3 Department of Child and Adolescent Psychiatry, Innlandet Hospital Trust, Postbox 990, Lillehammer, 2629 Norway; 4 Department of Psychology, Inland Norway University of Applied Sciences, Postbox 400, Elverum, 2418 Norway

**Keywords:** ADHD, Adults, Children, Drug utilization, Norway, Register-Study

## Abstract

**Background:**

The use of medication for Attention-Deficit/Hyperactivity Disorder (ADHD) increased globally throughout the early 2000s. This study examine trends in prevalences and incidences of medication use in Norway from 2006 to 2022.

**Methods:**

Data from the Norwegian Prescription Database were used to present one-year-prevalence and incidence rates of ADHD medication (ATC-group N06BA and C02AC02) for the overall population (ages 6–64) and within sex and age subgroups of children (ages 6–17) and adults (ages 18–64). Incident use was defined as the dispensing of medication, with no recorded use in the previous two calendar years.

**Results:**

The overall prevalence of ADHD medication use in 6- to 64-year-olds increased from 5.2 to 19.4 per 1000 in the period, most pronounced from 2020 and onwards. While males experienced a nearly threefold increase in use (from 7.3 to 20.6 per 1000), the use among females increased almost sixfold during the study period (from 3.0 to 18.1 per 1000). Consequently, the male-to-female prevalence-ratio decreased from 2.4 to 1. Children exhibited a higher prevalence of use compared to adults throughout the period, although the largest relative increase was observed in adults, particularly in female adults. In children the male-to-female ratio decreased from 3.2 to 2.0, primarily due to an increasing use in 13–17-year-old females. Among adults, prevalences were similar across most age groups, with the highest rates observed among those aged 18–24, where female use exceeded male use by the end of the period. The male-to-female prevalence-ratio in adults decreased from 1.6 to 0.9 during the period. Parallel to prevalent use, overall incident use increased from 1.4 to 5.0 per 1000 during the period, with the most pronounced increase occurring from 2020 and onwards. From this point, incident use among females aged 13–17, 18–24, and 25–34, exceeded that of males. The male-to-female incidence-ratio decreased from 1.8 to 0.9. The overall incidence to prevalence ratio remained similar throughout the period, being 0.27 in 2006 and 0.25 in 2022.

**Conclusion:**

A sustained increase in the prevalence of ADHD medication use was observed, with the most pronounced rise occurring among females and adults from 2020 and onwards. By 2022, the overall gender disparity in ADHD medication use had diminished, which should be considered in the context of a steep increase in incident use among adolescent and young adult females starting from 2020.

**Supplementary Information:**

The online version contains supplementary material available at 10.1186/s12888-024-06199-9.

## Background

Attention-Deficit/Hyperactivity Disorder (ADHD) is one of the most common neurodevelopmental disorders affecting 5.9% of children and 2.5% of adults [1]. Guidelines for the assessment and treatment of ADHD recommend the use of stimulants, such as methylphenidate and amphetamines, and non-stimulants, such as atomoxetine, when drug therapy is deemed appropriate [2, 3]. Studies on risks and benefits of drug-treatment emphasize that medication treatment for ADHD can lead to reductions in both symptom severity and functional impairment [4].

The worldwide prevalence of ADHD medication use increased throughout the early 2000s, including the Scandinavian countries [5]. Patterns across sex, age and geography indicated that males were more likely to use medication than females during childhood and adolescence, children were more frequently medicated than adults, and males initiated treatment earlier than females [6–12]. However, several studies investigating post-2010 patterns indicate that the level of ADHD medication use in children appeared to be stabilizing or even decreasing in some countries, with male-to-female-ratios converging and medication use increasing among older adolescents and adults [6, 7, 13–18]. One study presenting recent trends in ADHD medication use among Scandinavian children and adolescents aged 5 to 19 demonstrated an increase in ADHD medication use from 2010 to 2020, most notably in Sweden with a relative increase of 119% (reaching 35 users per 1000 inhabitants in 2020) followed by 38% relative increase in Denmark and 16% increase in Norway (reaching 22 per 1000 in 2020 in both countries) [19].

In the last two decades, more drugs have become available for the management of ADHD, and national guidelines for the evaluation and treatment of ADHD in children and adults are continuously updated [2]. Additionally, there have been several targeted economic and political efforts in Norway aimed at improving mental healthcare services, including ADHD in adults [20]. With changing guidelines, drug treatment options and mental health services, there is a need to monitor consequent drug utilization patterns.

Most Scandinavian studies, however, have focused on trends in ADHD medication use in children rather than adults. Furthermore, the increasing prevalence of ADHD medication use may be explained by the rising number of new users or by the duration of treatment. To date, no information is available in the literature on trends in incident ADHD medication use, and how it relates to changes in prevalent use, in a Scandinavian country.

Therefore, the aim of this study was to present and compare prevalence and incidence trends in ADHD medication use in the Norwegian population among individuals aged 6–64 years. Trends in use are presented as one-year prevalence and incidence rated, stratified by age groups and sex, for the period 2006 to 2022.

## Methods

### Data source

This study utilizes data from the Norwegian Prescription Database (NorPD) [21, 22]. The NorPD has recorded information on all prescription drugs dispensed to outpatients at Norwegian pharmacies since 2004. The database includes detailed information about the patient, the prescriber, the pharmacy, and the dispensed drug. Drug information is classified according to the Anatomical Therapeutic Chemical (ATC) classification system [23].

### Study population and study medication

All individuals who filled at least one prescription for ADHD medication between January 1, 2006 and December 31, 2022, were included in the study. Due to a very low prevalence of use among patients below 6 and above 64 years of age, only individuals aged 6 to 64 years with a valid personal identification number were included.

The study medication comprised drugs licensed for ADHD treatment during the study period, including centrally acting stimulants or sympathomimetics (ATC group N06BA) and guanfacine (ATC code C02AC02).

Modafinil (ATC code N06BA07) was excluded from both prevalence and incidence calculations as its main indication is for excessive daytime sleepiness. In this study, dispensed ADHD drugs is used as a proxy for the use of ADHD medications as a group (use of any ADHD drug).

### Analytical approach

One-year prevalence of ADHD medication use was defined as the total number of individuals who were dispensed at least one ADHD medication during a given year per thousand inhabitants as of January 1 each year from 2006 to 2022.

An individual was considered an incident user if they were dispensed an ADHD medication a specific year with no dispensations in the previous two calendar years. Incidence was calculated by dividing the number of incident users each year by the total number of inhabitants at risk on January 1 each year from 2006 to 2022. Inhabitants “at risk” was the total number of inhabitants on January 1 of a given year, minus those who had ADHD medication dispensed at least once in the previous two calendar years.

One-year prevalence and incidence of ADHD medication use were calculated overall (for individuals aged 6 to 64 years) and according to age groups and sex. Age groups comparable to previous studies on trends in ADHD medication use in the Norwegian population were used [9, 16]; children ( 6–12, 13–17) and adults (18–24, 25–34, 35–44 and 45–64).

Additionally, by dividing incidence by prevalence, incidence-to-prevalence ratios were calculated to study the relationship- between incidence and prevalence over time.

Prevalent and incident use of ADHD medication were described in terms of proportions (per 1000), and 95% confidence intervals (CI) for proportions were calculated using the continuity corrected version of the score [24]. 95% CI were compared to assess overall changes over time in terms of statistical significance. In cases where the 95% CIs around a value do not overlap, it indicates that the difference is statistically significant (*p* < 0.05).

### Ethical considerations

The data used for this study are anonymous and, approval from ethical committees was not needed. Patient consent is not needed according to Norwegian health register act.

## Results

### Trends in prevalence of ADHD medication use

The overall prevalence of ADHD medication use among individuals aged 6 to 64 years increased from 5.2 (95% CI: 5.1–5.2) per 1000 in 2006 to 19.4 (95% CI: 19.3–19.5) per 1000 in 2022 (Supplementary Fig. 1). While males experienced a nearly threefold increase in use (from 7.3 (95% CI: 7.2–7.4) per 1000 to 20.6 (95% CI: 20.4–20.8) per 1000), use among females increased almost sixfold in the study period (from 3.0 (95% CI: 2.9–3.1) per 1000 to 18.1 (95% CI: 17.9–19.3) per 1000). Consequently, the male-to-female prevalence ratio decreased from 2.4 to 1.1 during this period (Supplementary Table 1).

**Fig. 1:**
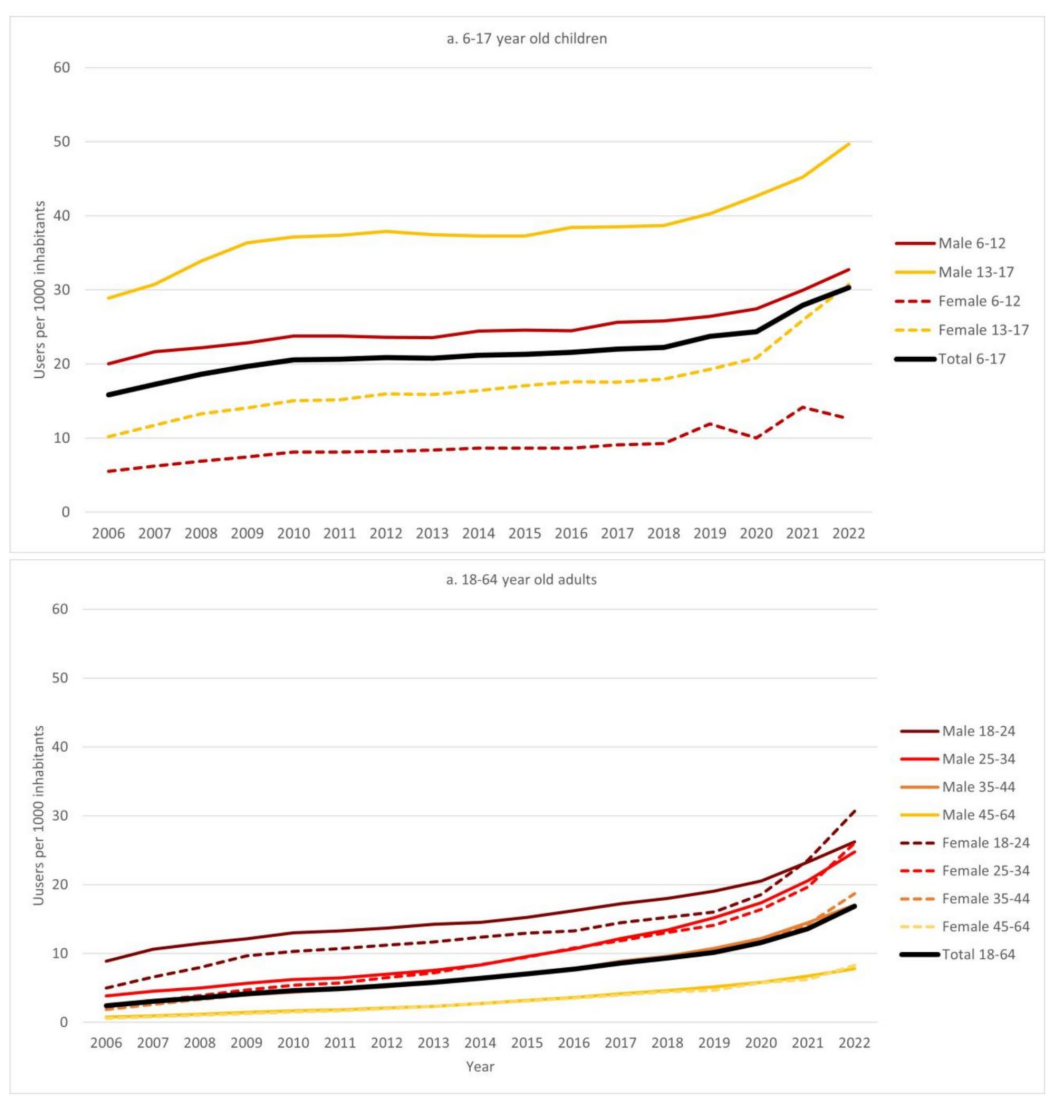
One-year prevalence (users per 1000 inhabitants) of ADHD medication use in 6–17-year-old children **(a)** and 18–64-year-old adults **(b)** in Norway in the period 2006 to 2022

The overall use of ADHD medication among children aged 6–17 years increased from 15.9 (95% CI: 15.6–16.1) to 30.3 (95% CI: 30.0-30.7) per 1000 during the study period (Fig. 1a). Boys in the age groups 6–12 years and 13–17 years consistently exhibited higher levels of ADHD medication use compared to girls in the same age groups throughout the study period. The highest level of use was observed in 2022 among boys aged 13–17 years (49.7 per 1000). The overall trend showed a modest yearly increase up to 2010, followed by a plateau until 2020, after which a large increase in use was observed across all sub-groups. This increase was more pronounced among girls, as indicated by the male-to-female prevalence ratio decreasing from 3.2 in 2006 to 2.0 in 2022 (Supplementary Table 1).

Among adults aged 18–64, the overall use of ADHD medication increased from 2.4 (95% CI: 2.3–2.5) to 16.9 (95% CI: 16.7–17.3) per 1000 during the study period (Fig. 1b). There was a steady increase up to approximately 2020, followed by a marked increase in all sub-groups. The increase was most prominent among females, who experienced nearly a tenfold rise (1.9 per 1000 to 17.6 per 1000). Throughout the study period, levels of use were relatively similar between males and females in corresponding age groups, except for those aged 18–24, where lower use was observed among females until 2020, after which female use exceeded that of males in 2022. The level of use decreased with increasing age for both sexes.

### Trends in incident use of ADHD medication

The overall incidence of ADHD medication use among individuals aged 6 to 64 years increased from 1.4 (95% CI: 1.3–1.4) per 1000 in 2006 to 5.0 (95% CI: 4.9–5.1) per 1000 in 2022 (Supplementary Fig. 2). While a nearly threefold increase in incident use was observed among males (from 1.8 (95% CI: 1.7–1.9) per 1000 to 4.8 (95% CI: 4.7–4.9) per 1000), there was a nearly fivefold increase among females in the study period (from 1.0 (95% CI: 1.0-1.1) per 1000 to 5.2 (95% CI: 5.1–5.3) per 1000). Consequently, the male-to-female incidence ratio decreased from 1.8 in 2006 to 0.9 in 2022 (Supplementary Table 2).

**Fig. 2:**
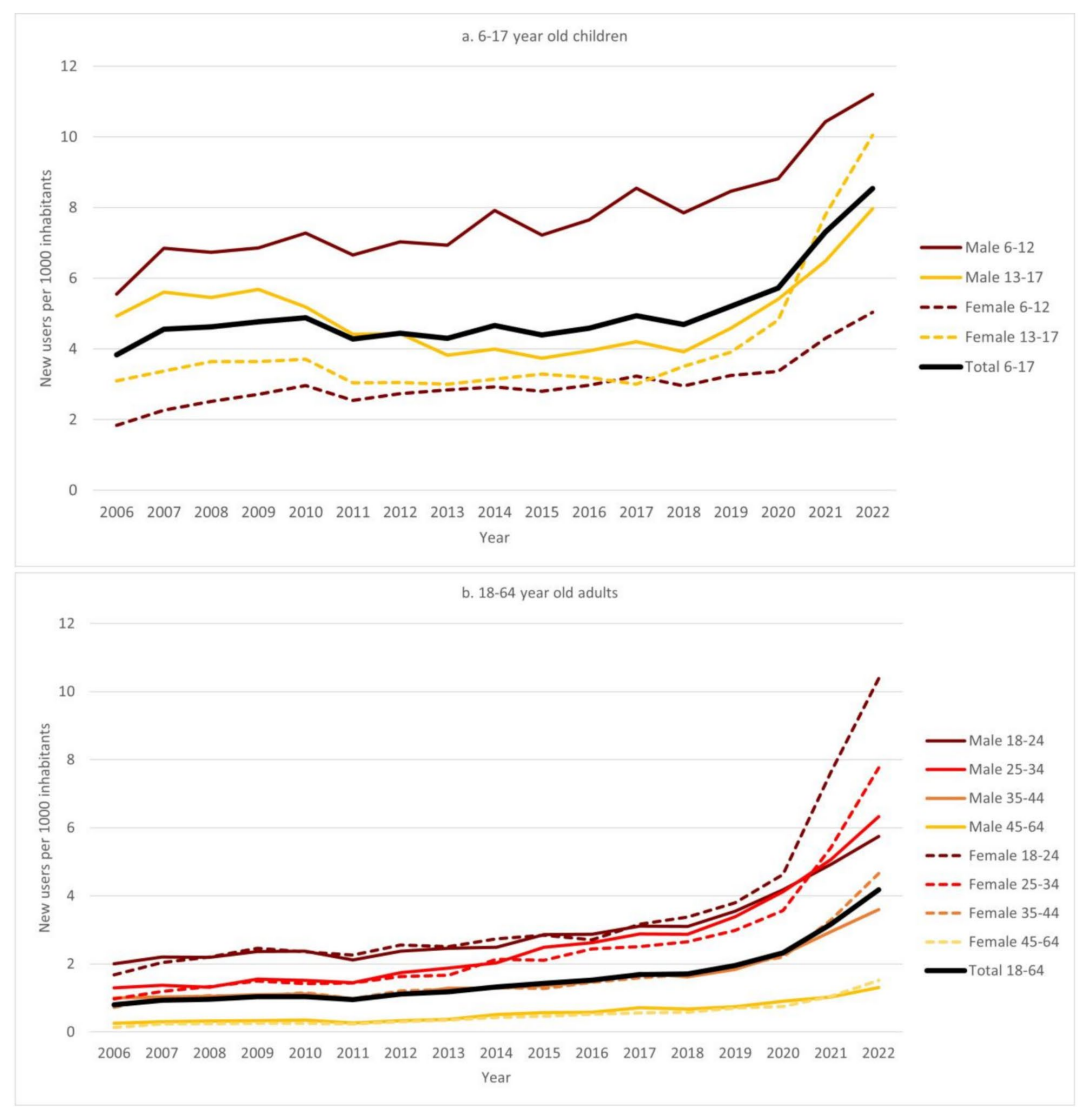
One-year incidence (new users per 1000 inhabitants) of ADHD medication use in 6 -17-year-old children **(a)** and 18-64-year-old adults **(b)** in Norway in the period 2006 to 2022

The overall incident use of ADHD medication among children aged 6 to 17 years increased from 3.8 (95% CI: 3.7-4.0) per 1000 to 8.5 (95% CI: 8.3–8.7) per 1000 in the study period (Fig. 2a). All subgroups showed an increase up to 2010, followed by stability until 2020, after which a large increase was observed across all sub-groups. In 2022, incident use among males aged 6 to 12 years was more than double that of females (11.2 per 1000 and 5.0 per 1000, respectively). Among 13 to 17-year-olds, incident use was lower among females than males up to 2020, but by 2022 incident use among females exceeded that of males (10.0 per 1000 for females and 8.0 per 1000 for males). The male-to-female incidence ratio decreased from 3.0 to 2.2 in 6-12-year-olds and from 1.6 to 0.8 in 13-17-year-olds (Supplementary Table 2).

Among adults aged 18–64 years, the overall incidence of ADHD medication use increased from 0.8 (95% CI: 0.7–0.9) per 1000 to 4.2 (95% CI: 4.1–4.2) per 1000 in the study period (Fig. 2b). There was a stable increase in incident use up to 2020, followed by a pronounced increase across all subgroups. Incidence levels were comparable between males and females in the oldest age groups, whereas among 18 to 24 and 25 to 34-year-olds, females exceeded males by the end of the study period. The highest level of incident use was observed among females aged 18 to 24 in 2022 (10.4 per 1000). The overall male-to-female incidence ratio was 0.9 in 2022 (Supplementary Table 2).

### Trends in the incidence-to-prevalence ratio of ADHD medication use

**Fig. 3:**
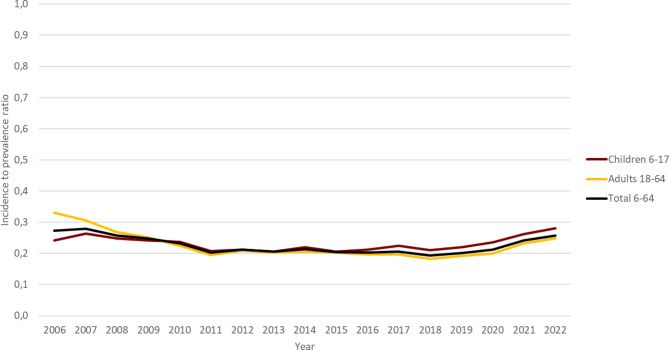
Incidence to prevalence ratios of ADHD medication use in in 6 -64-year-old children and adults in Norway in the period 2006 to 2022

The incidence-to-prevalence ratio among individuals aged 6 to 64 years was 0.27 in 2006 and 0.26 in 2022, with ratios around 0.20 during the period between 2011 and 2020 (Fig. 3). Similar patterns were observed among children aged 6 to 17 years and adults aged 18 to 64 years. The incidence-to-prevalence ratio among children aged 6 to 17 years exceeded that of adults aged 18 to 64 years throughout the period and was 0.28 in 2022 (0.25 in adults).

## Discussion

Both the prevalence and incidence of ADHD medication use among Norwegians aged 6 to 64 years increased fourfold from 2006 to 2022, primarily due to a relative increase in females and adults. Consequently, the male-to-female prevalence ratio decreased in the period and was close to 1 in 2022. This trend must be considered in the context of incident use among adolescent and young adult females, which exceeded and was higher than in males at the end of the study period.

### Trends in ADHD medication use in children

Among children aged 6 to 17 years, there was a twofold increase in prevalence and incidence of use during the study period. As previously shown, the level of use stabilized in the middle of the study period (around 2010–2020) [9, 19]. However, since then, there has been a steep increase in use among both boys and girls, most prominently from 2020 and onwards, coinciding with the COVID-19 pandemic. Parallel, a report from the Norwegian Institute of Public Health indicates an increase in ADHD diagnoses among children and adolescents, particularly during the period from 2020 to 2022 [25]. The increase is most pronounced among teenage girls. Our findings align with other Scandinavian studies documenting the increasing use of psychotropic drugs in children during the pandemic, most notably among those aged 12 to 17 years in Denmark [26, 27].

The prevalence of use remained higher among boys compared to girls in 2022. However, the male-to-female prevalence ratio decreased from 3.2 to 2.0 during the period, which may be explained by the incidence among 13 to 17-year-old girls exceeding that of boys by the end of the study period. A similar trend has recently been observed in Finland [28]. The observed shift in male to female ratio indicates an increased recognition and/or pharmacological treatment of ADHD in girls.

### Trends in ADHD medication use in adults

Among adults aged 18–64 years, there was an eightfold relative increase in prevalence and a fivefold increase in incident use during the period, with the most pronounced increases observed in in females and from 2020 and onwards. While boys still exhibit higher levels of ADHD medication use to girls in the child population, young female adults aged 18–34 years surpassed males in both prevalence and incidence of use by the end of the study period.

As a result, adult females constitute an increasingly larger proportion of all individuals on ADHD medication in Norway, contributing to the overall male-to-female prevalence ratio in 6-64-year-olds which was close to 1 in 2022. Parallel, a report from the Norwegian Institute of Public Health on adult mental health indicates an increase in ADHD diagnoses among adults in Norway [29]. The increase is particularly noticeable among young adults aged 18 to 27 years.

The observed trend of increasing ADHD medication use in Norwegian adults align with findings from the US, Germany and Australia [15, 30, 31]. In the US, as in Norway, there was a substantial increase in adult ADHD medication use during the COVID-19 pandemic starting in 2020.

The increase in incident and prevalent use could result from increased recognition of ADHD and the subsequent need for ADHD medication treatment in adulthood, particularly among females. A meta-analysis of longitudinal studies showed that up to one-third of childhood ADHD cases continued to meet full diagnostic criteria into their 20s, with around 65% continuing to experience impairing symptoms [32]. Some studies also indicate a growing number of adults (and children) being diagnosed with ADHD [18, 33], suggesting an increase in patients’ need for continued and/or initiation of ADHD medication treatment.

Another explanation could be that guidelines and diagnostic criteria have been modified in a way that promotes the diagnosis of ADHD in adults. The updated Norwegian guidelines for diagnosing and treating ADHD specifically recommend the use of Diagnostic and Statistical Manual of Mental Disorders, Fifth Edition (DSM-V) in the diagnostic process, compared to earlier versions were a more flexible use of both DSM-IV/V and International Classification of Diseases, 10th Revision (ICD-10) was acknowledged [2]. As summarized and argued by Epstein and Loren [34], changes in the DSM-V diagnostic criteria made the ADHD criteria more applicable to adolescents and adults. For instance, in DSM-V the age of onset of symptoms required is before 12 years, compared to before seven years of age in ICD-10 and in DSM-IV [35–37], making adult self-reporting of symptoms more pertinent. Thus, the observed increase in ADHD medication use in adults may reflect both an adaption to new guidelines and diagnostic criteria in clinical practice.

### Increasing prevalences of ADHD medication use – more new users or increasing duration of use?

Increasing prevalences of use can be explained by both increasing number of new users, and longer treatment duration once ADHD medication is initiated. The incidence-to-prevalence ratio in the total study population of 6-64-year-olds was relatively stable around 0.2 most of the study period but increased to 0.25 at the end of the study period. Thus, in 2022 around 25% of the increase in ADHD drug use can be attributed to new users’ contribution to prevalent use, and this contribution seems to have remained unchanged over time. On the other hand, the remaining 75% of the increase in prevalence of use is attributed to longer duration of treatment in patients already initiated on ADHD drugs. During the study period, several medication alternatives have been licensed for use. In general, long-acting formulations are associated with longer treatment duration than short-acting formulations [38]. A study by Karlstad et al. showed that the use of long-acting methylphenidate increased among adults in the time period 2008 to 2011 compared to short-acting formulations [16]. It is possible that more diversified treatment options and use of medication that is associated with improved persistence of drug use over time may also explain the increase in prevalences in overall use of ADHD medication.

The prevalence-to-incidence ratio was higher in children aged 6–17 years than in adults aged 18–64 years. Thus, new users accounted for a larger part of increasing prevalence of use in children than in adults, which may be a logical consequence of continuation of treatment over time from childhood into older age groups.

### Strengths and limitations

This study is based on data from a nationwide prescription register covering the entire population of Norway, which eliminates selection and recall bias. However, our results may slightly underestimate ADHD drug use, as the NorPD does not include information on drugs administered to individuals in hospitals or institutions. This study examines ADHD medication use as a group and reveals no information on trends in use of specific ADHD drug substances. Additionally, the analyses were based on filled prescriptions as proxies for the use of ADHD medication, and it is unknown whether the patients actually consumed the medication. No information was available on the indication of use. Furthermore, we do not know to what extent some of the increase in medication use was for other indications, such as narcolepsy and/or excessive daytime sleepiness, as some centrally acting stimulants other than modafinil may have been used for this purpose [39]. However, due to the rarity of this condition [40], we consider the impact on the results as negligible.

## Conclusion

From 2006 to 2022, substantial changes have occurred in the use of ADHD medication in Norway. While young males continue to exhibit the highest usage, significant shifts have occurred in the consumption patterns among young females. Consequently, gender disparities in ADHD medication use among individuals aged 6 to 64 years were no longer evident by 2022. Notably, the use among the elderly is currently experiencing a relatively greater increase compared to that among the younger population. The observed changes in trends in use, particularly from 2020, emphasize the continued need to monitor medication use in a field where new knowledge arises, new drug-treatments are licensed, and guidelines are being updated.

## Electronic supplementary material

Below is the link to the electronic supplementary material.

Supplementary Material 1

## Data Availability

This study utilizes data from the Norwegian Prescription Database (NorPD), and aggregated data were retrieved from the register holder.

## Abbreviations

ADHD: Attention-Deficit/Hyperactivity Disorder
NorPD: Norwegian Prescription Database
ATC classification system: Anatomical Therapeutic Chemical classification system
DSM-V: Diagnostic and Statistical Manual of Mental Disorders, Fifth Edition
ICD-10: International Classification of Diseases, 10th Revision

## References

[1] Faraone SV, Banaschewski T, Coghill D, Zheng Y, Biederman J, Bellgrove MA, et al. The World Federation of ADHD International Consensus Statement: 208 evidence-based conclusions about the disorder. Neurosci Biobehav Rev. 2021. 10.1016/j.neubiorev.2021.01.022. 33549739 10.1016/j.neubiorev.2021.01.022 PMC8328933

[2] Norwegian Directorate of Health. National guidelines for diagnosis and managment of ADHD/Hyperkinetic disorder. (updated May 4th, 2022). https://www.helsedirektoratet.no/retningslinjer/adhd. Accessed December 15, 2023.

[3] National Institute for Health and Care Excellence (NICE). NICE guideline [NG87]: Attention deficit hyperactivity disorder: diagnosis and management (updated January 2023). https://www.nice.org.uk/guidance/NG87. Accessed February 3rd, 2024. 29634174

[4] Chang Z, Ghirardi L, Quinn PD, Asherson P, D’Onofrio BM, Larsson H. Risks and benefits of attention-deficit/hyperactivity disorder medication on behavioral and neuropsychiatric outcomes: a qualitative review of pharmacoepidemiology studies using linked prescription databases. Biol Psychiatry. 2019;86(5):335–43. 31155139 10.1016/j.biopsych.2019.04.009 PMC6697582

[5] Raman SR, Man KKC, Bahmanyar S, Berard A, Bilder S, Boukhris T, et al. Trends in attention-deficit hyperactivity disorder medication use: a retrospective observational study using population-based databases. Lancet Psychiatry. 2018;5(10):824–35. 30220514 10.1016/S2215-0366(18)30293-1

[6] Bachmann CJ, Wijlaars LP, Kalverdijk LJ, Burcu M, Glaeske G, Schuiling-Veninga CCM, et al. Trends in ADHD medication use in children and adolescents in five western countries, 2005–2012. Eur Neuropsychopharmacol. 2017;27(5):484–93. 28336088 10.1016/j.euroneuro.2017.03.002

[7] Board AR, Guy G, Jones CM, Hoots B. Trends in stimulant dispensing by age, sex, state of residence, and prescriber specialty - United States, 2014–2019. Drug Alcohol Depend. 2020;217:108297. 32961454 10.1016/j.drugalcdep.2020.108297 PMC7851748

[8] Derks EM, Hudziak JJ, Boomsma DI. Why more boys than girls with ADHD receive treatment: a study of Dutch twins. Twin Res Hum Genet. 2007;10(5):765–70. 17903118 10.1375/twin.10.5.765

[9] Furu K, Karlstad Ø, Zoega H, Martikainen JE, Bahmanyar S, Kieler H, et al. Utilization of stimulants and Atomoxetine for Attention-Deficit/Hyperactivity disorder among 5.4 million children using Population-based Longitudinal Data. Basic Clin Pharmacol Toxicol. 2017;120(4):373–9. 27911044 10.1111/bcpt.12724

[10] Kok FM, Groen Y, Fuermaier ABM, Tucha O. The female side of pharmacotherapy for ADHD-A systematic literature review. PLoS ONE. 2020;15(9):e0239257. 32946507 10.1371/journal.pone.0239257 PMC7500607

[11] Lillemoen PK, Kjosavik SR, Hunskar S, Ruths S. Prescriptions for ADHD medication, 2004-08. Tidsskr nor Laegeforen. 2012;132(16):1856–60. 22986969 10.4045/tidsskr.11.1270

[12] Zetterqvist J, Asherson P, Halldner L, Langstrom N, Larsson H. Stimulant and non-stimulant attention deficit/hyperactivity disorder drug use: total population study of trends and discontinuation patterns 2006–2009. Acta Psychiatr Scand. 2013;128(1):70–7. 22943458 10.1111/acps.12004

[13] Akmatov MK, Holstiege J, Batzing J. Secular trends and regional variations in pharmacotherapy of attention-deficit/hyperactivity disorder (ADHD) among children and adolescents in Germany. BMC Psychiatry. 2021;21(1):405. 34391396 10.1186/s12888-021-03409-6 PMC8364007

[14] Beau-Lejdstrom R, Douglas I, Evans SJ, Smeeth L. Latest trends in ADHD drug prescribing patterns in children in the UK: prevalence, incidence and persistence. BMJ Open. 2016;6(6):e010508. 27297009 10.1136/bmjopen-2015-010508 PMC4932306

[15] Grimmsmann T, Himmel W. The 10-year trend in drug prescriptions for attention-deficit/hyperactivity disorder (ADHD) in Germany. Eur J Clin Pharmacol. 2021;77(1):107–15. 32803292 10.1007/s00228-020-02948-3 PMC7782395

[16] Karlstad Ø, Zoëga H, Furu K, Bahmanyar S, Martikainen JE, Kieler H, et al. Use of drugs for ADHD among adults—a multinational study among 15.8 million adults in the nordic countries. Eur J Clin Pharmacol. 2016;72(12):1507–14. 27586399 10.1007/s00228-016-2125-y PMC5110707

[17] Morkem R, Patten S, Queenan J, Barber D. Recent trends in the prescribing of ADHD medications in Canadian primary care. J Atten Disord. 2020;24(2):301–8. 28748725 10.1177/1087054717720719

[18] Renoux C, Shin JY, Dell’Aniello S, Fergusson E, Suissa S. Prescribing trends of attention-deficit hyperactivity disorder (ADHD) medications in UK primary care, 1995–2015. Br J Clin Pharmacol. 2016;82(3):858–68. 27145886 10.1111/bcp.13000 PMC5338115

[19] Sorensen AMS, Wesselhoeft R, Andersen JH, Reutfors J, Cesta CE, Furu K, et al. Trends in use of attention deficit hyperactivity disorder medication among children and adolescents in Scandinavia in 2010–2020. Eur Child Adolesc Psychiatry. 2023;32(10):2049–56. 35831669 10.1007/s00787-022-02034-2

[20] Norwegian Ministry of Health And Caring Services. Action Plan for Mental Health (2023–2033) (in Norwegian: Opptrappingsplan for psykisk helse). Parlamentary White Paper 23 (2022–2023) (First published 1999). https://www.regjeringen.no/contentassets/0fb8e2f8f1ff4d40a522e3775a8b22bc/no/pdfs/stm202220230023000dddpdfs.pdf. Accessed November 15, 2023.

[21] The Norwegian Prescription Database. https://www.fhi.no/en/he/norpd/norwegian-prescription-database/. Accessed January 15, 2024.

[22] Furu K. Establishment of the nationwide Norwegian prescription database (NorPD)-new opportunities for research in pharmacoepidemiology in Norway. Nor J Epidemiol. 2008;18(2):129–36.

[23] WHO Collaborating Centre for Drug Statistics Methodology. ATC/DDD methodology. https://atcddd.fhi.no/atc_ddd_methodology/purpose_of_the_atc_ddd_system/. Accessed November 15, 2023.

[24] Vollset SE. Confidence intervals for a binomial proportion. Stat Med. 1993;12(9):809–24. 8327801 10.1002/sim.4780120902

[25] Bang L, Furu K, Handal M, Torgersen L, Støle HS, Suren P et al. Public Health Report: Mental distress and disorders among children and adolescents. Norwegian Institute of Public Health, 2023. https://www.fhi.no/he/folkehelserapporten/psykisk-helse/psykisk-helse-hos-barn-og-unge/?term=. Accessed August 29, 2024.

[26] Bliddal M, Rasmussen L, Andersen JH, Jensen PB, Pottegard A, Munk-Olsen T, et al. Psychotropic medication Use and Psychiatric disorders during the COVID-19 pandemic among Danish children, adolescents, and young adults. JAMA Psychiatry. 2023;80(2):176–80. 36515919 10.1001/jamapsychiatry.2022.4165 PMC9856810

[27] Kuitunen I. Psychotropic medication use in pediatric population during COVID-19 pandemic. Acta Psychiatr Scand. 2022;146(4):381–3. 35894545 10.1111/acps.13483 PMC9353282

[28] Vuori M, Koski-Pirilä A, Martikainen JE, Saastamoinen L. Gender- and age-stratified analyses of ADHD medication use in children and adolescents in Finland using population-based longitudinal data, 2008–2018. Scand J Public Health. 2020:1403494820901426. 10.1177/1403494820901426 PMC7346711 31985349

[29] Tesli MS, Kirkøen B, Handal M, Torvik FA, Odsbu I, Knudsen AKS. Public Health Report: Mental distress and disorders in adults.: The Norwegian Institute of Public Health, 2024. https://www.fhi.no/he/folkehelserapporten/psykisk-helse/psykiske-lidelser-voksne/?term=. Accessed August 28, 2024.

[30] Bruno C, Havard A, Gillies MB, Coghill D, Brett J, Guastella AJ, et al. Patterns of attention deficit hyperactivity disorder medicine use in the era of new non-stimulant medicines: a population-based study among Australian children and adults (2013–2020). Aust N Z J Psychiatry. 2023;57(5):675–85. 35999695 10.1177/00048674221114782

[31] Danielson ML, Bohm MK, Newsome K, Claussen AH, Kaminski JW, Grosse SD, et al. Trends in stimulant prescription fills among commercially insured children and adults - United States, 2016–2021. MMWR Morb Mortal Wkly Rep. 2023;72(13):327–32. 36995976 10.15585/mmwr.mm7213a1 PMC10078845

[32] Faraone SV, Biederman J, Mick E. The age-dependent decline of attention deficit hyperactivity disorder: a meta-analysis of follow-up studies. Psychol Med. 2006;36(2):159–65. 16420712 10.1017/S003329170500471X

[33] Davidovitch M, Koren G, Fund N, Shrem M, Porath A. Challenges in defining the rates of ADHD diagnosis and treatment: trends over the last decade. BMC Pediatr. 2017;17(1):218. 29284437 10.1186/s12887-017-0971-0 PMC5747128

[34] Epstein JN, Loren RE. Changes in the definition of ADHD in DSM-5: subtle but important. Neuropsychiatry (London). 2013;3(5):455–8. 24644516 10.2217/npy.13.59 PMC3955126

[35] American Psychiatric Association. The Diagnostic and Statistical Manual of Mental Disorders, Fifth Edition, 2013.

[36] American Psychiatric Association. The Diagnostic and Statistical Manual of Mental Disorders, Fourth Edition, 1994.

[37] World Health Organization. International Classification of Diseases, 10th revision, 1993.

[38] Gajria K, Lu M, Sikirica V, Greven P, Zhong Y, Qin P, et al. Adherence, persistence, and medication discontinuation in patients with attention-deficit/hyperactivity disorder - a systematic literature review. Neuropsychiatr Dis Treat. 2014;10:1543–69. 25187718 10.2147/NDT.S65721 PMC4149449

[39] The Norwegian Medicines Manual for Health Personnel. (Norsk Legemiddelhåndbok), Therapy of narcolepsy: https://www.legemiddelhandboka.no/T6.8.1/Narkolepsi. Accessed 10th April 2024.

[40] Ohayon MM, Priest RG, Zulley J, Smirne S, Paiva T. Prevalence of narcolepsy symptomatology and diagnosis in the European general population. Neurology. 2002;58(12):1826–33. 12084885 10.1212/wnl.58.12.1826

