# Supplementary material for: Nationwide trends in the use of ADHD medications in the period 2006–2022: a study from the Norwegian prescription database
Source: BMC Psychiatry. 2024 Nov 5;24:767. doi: 10.1186/s12888-024-06199-9 (PMC11539614; doi:10.1186/s12888-024-06199-9)
Supplement: Supplementary file 1 — Supplementary Material 1 [file 12888_2024_6199_MOESM1_ESM.docx]

## **Supplementary figures and tables**

### Supplementary figure 1. One-year prevalence (users per 1000 inhabitants) of ADHD medication use in 6- to 64-year-old individuals in Norway in the period 2006 to 2022


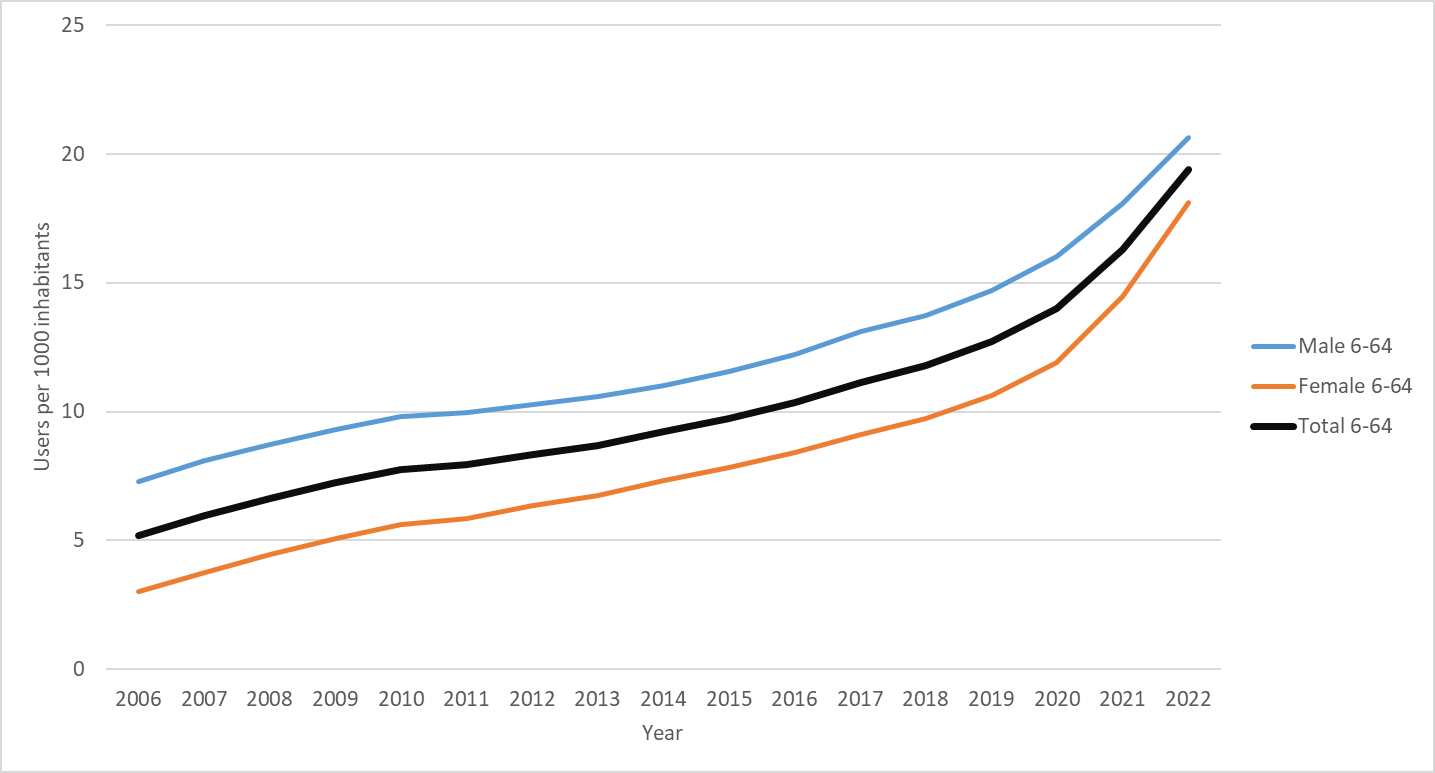


### Supplementary figure 2. One-year incidence (new users per 1000 inhabitants) of ADHD medication use in 6- to 64-year-old individuals in Norway in the period 2006 to 2022


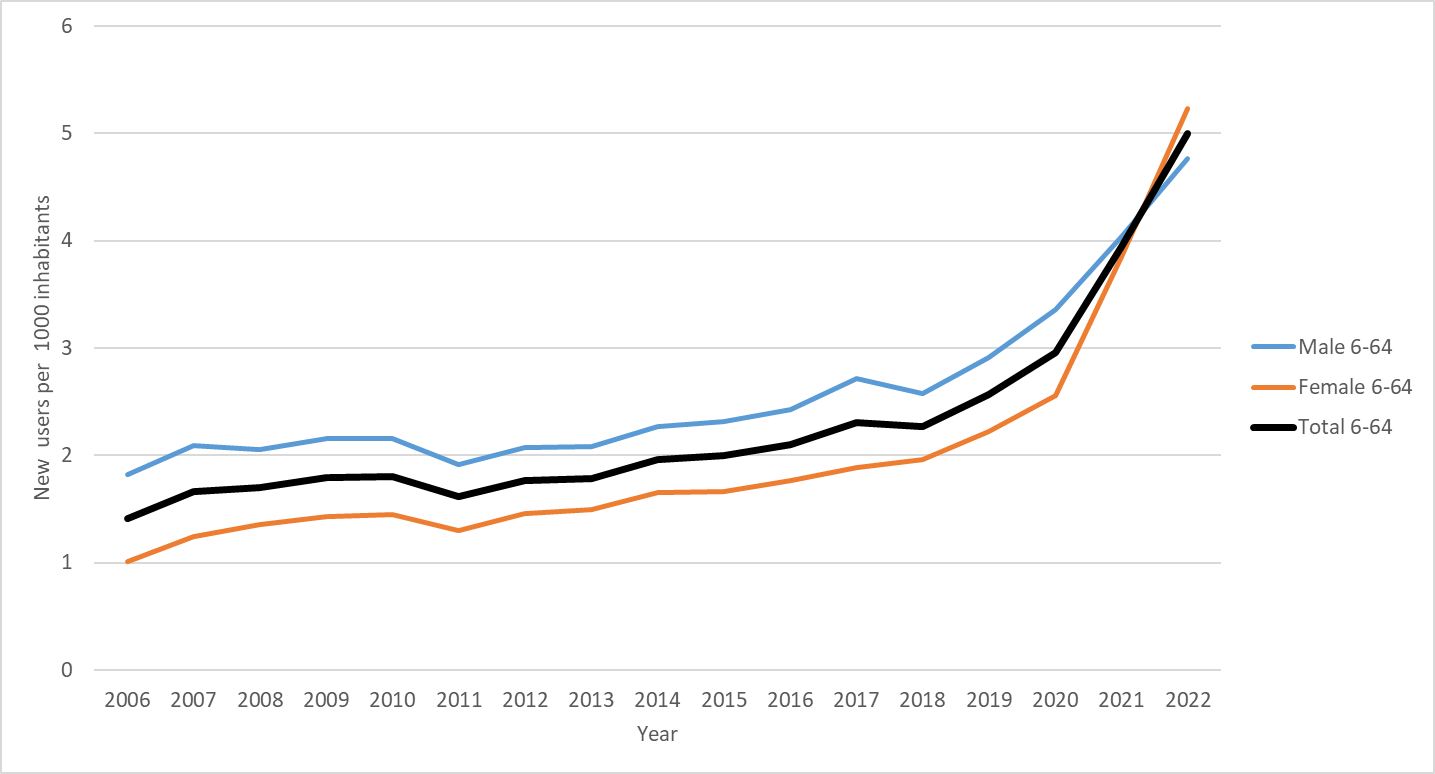


Supplementary Table 1. One-year male/female prevalence-ratios of ADHD medication use in 6-64 year olds in Norway in the period 2006-2022

### Supplementary Table 2. One-year male/female incidence-ratios of ADHD medication use in 6-64 year olds in Norway in the period 2006-2022
